# The Negative Effects of KPN00353 on Glycerol Kinase and Microaerobic 1,3-Propanediol Production in *Klebsiella pneumoniae*

**DOI:** 10.3389/fmicb.2017.02441

**Published:** 2017-12-07

**Authors:** Wen-Yih Jeng, Novaria S. D. Panjaitan, Yu-Tze Horng, Wen-Ting Chung, Chih-Ching Chien, Po-Chi Soo

**Affiliations:** ^1^University Center for Bioscience and Biotechnology, National Cheng Kung University, Tainan, Taiwan; ^2^Department of Biochemistry and Molecular Biology, National Cheng Kung University, Tainan, Taiwan; ^3^Institute of Medical Sciences, College of Medicine, Tzu Chi University, Hualien, Taiwan; ^4^Department of Laboratory Medicine and Biotechnology, College of Medicine, Tzu Chi University, Hualien, Taiwan; ^5^Graduate School of Biotechnology and Bioengineering, Yuan Ze University, Taoyuan, Taiwan

**Keywords:** 1, 3-propanediol, glycerol kinase, *Klebsiella pneumoniae*, carbohydrate phosphotransferase system, EIIA

## Abstract

1,3-Propanediol (1,3-PD) is a valuable chemical intermediate in the synthesis of polyesters, polyethers, and polyurethanes, which have applications in various products such as cloth, bottles, films, tarpaulins, canoes, foam seals, high-resilience foam seating, and surface coatings. *Klebsiella pneumoniae* can produce 1,3-PD from glycerol. In this study, KPN00353, an EIIA homologue in the phosphoenolpyruvate (PEP):carbohydrate phosphotransferase system (PTS), was found to play a negative regulatory role in 1,3-PD production under microaerobic conditions via binding to glycerol kinase (GlpK). The primary sequence of KPN00353 is similar to those of the fructose-mannitol EIIA (EII^Fru^ and EIIA^Mtl^) family. The interaction between KPN00353 and GlpK resulted in inhibition of the synthesis of glycerol-3-phosphate (G3P) and correlated with reductions in glycerol uptake and the production of 1,3-PD. Based on structure modeling, we conclude that residue H65 of KPN00353 plays an important role in the interaction with GlpK. We mutated this histidine residue to aspartate, glutamate, arginine and glutamine to assess the effects of each KPN00353 variant on the interaction with GlpK, on the synthesis of G3P and on the production of 1,3-PD. Our results illuminate the role of KPN00353 in 1,3-PD production by *K. pneumoniae* under microaerobic conditions.

## Introduction

Glycerol is a structural component of many lipids (glycerolipids) in organisms, and yeast can produce glycerol by the fermentation of sugar. Since ample glycerol occurs in nature, many microorganisms can utilize glycerol as a sole carbon and energy source (Wang et al., 2001). *Klebsiella pneumoniae* is not only an opportunistic pathogen but also a saprophytic microorganism that can be found in sewage, soil, plants, insects, animals and humans (Bagley, 1985; Medrano et al., 2014). Glycerol dissimilation in *K*. *pneumoniae* can be divided into aerobic metabolism (respiratory metabolism) and two branches of glycerol fermentation. In aerobic metabolism, the dissimilation of glycerol is begun by glycerol kinase (GlpK), which synthesizes glycerol-3-phosphate (G3P), followed by oxidation to dihydroxyacetone phosphate (DHAP). Glycerol fermentation by *Klebsiella pneumoniae* involves two parallel pathways: the reductive and oxidative pathways. The oxidative pathway leads to the production of DHAP. DHAP from either respiratory metabolism or oxidative fermentation is channeled to produce pyruvate. The reductive pathway leads to 1,3-propanediol (1,3-PD) production (Celinska, 2012; Kumar and Park, 2017). The 1,3-PD is a valuable chemical intermediate to produce polyesters, polyethers, polyurethanes and polytrimethylene terephthalate (PTT) (Zeng and Biebl, 2002). 1,3-PD can be produced through either chemical synthesis or microbial conversion. The chemical synthesis processes produce toxic byproducts and require a reduction step under high hydrogen pressure (Sullivan, 1993). In contrast, biosynthesis is a comparatively attractive option because it can use renewable feedstock and does not generate a toxic intermediate. 1,3-PD is a product of glycerol fermentation by *Klebsiella, Enterobacter, Citrobacter, Lactobacillus*, and *Clostridium* species and by engineered *Escherichia coli* (Huang et al., 2002; Saxena et al., 2009; Pyne et al., 2016). Among these organisms, *Clostridium butyricum* and *Klebsiella pneumoniae* are considered the best natural 1,3-PD producers (Saxena et al., 2009). However, *K. pneumoniae* attracts substantial attention because of the availability of genetic engineering tools that are applicable to *K. pneumoniae* but not *C. butyricum* (Celinska, 2012; Kumar and Park, 2017). *K. pneumoniae* can produce 1,3-PD from glycerol under anaerobic, aerobic or microaerobic conditions (Forage and Lin, 1982; Huang et al., 2002; Chen X. et al., 2003; Saxena et al., 2009), but the microaerobic conditions are better than the anaerobic and aerobic conditions (Chen X. et al., 2003). In our previous study, the amount of 1,3-PD produced by *K. pneumoniae* was increased by the overexpression of 1,3-propanediol oxidoreductase (PDOR) or both PDOR and DhaB under microaerobic conditions (Horng et al., 2010).

The bacterial phosphoenolpyruvate (PEP):carbohydrate phosphotransferase system (PTS) transports and phosphorylates carbohydrates, such as hexoses (e.g., glucose and fructose), sugar alcohols (e.g., mannitol), amino sugars (e.g., *N*-acetylglucosamine), disaccharides (e.g., cellobiose) and other carbon sources (Al Makishah and Mitchell, 2013; Deutscher et al., 2014; Liang et al., 2015). The PTS is a phosphorylation cascade usually composed of one membrane-spanning protein/domain and four soluble proteins/domains that sequentially transfer phosphate from PEP to the substrate. Enzyme I (EI) and histidine protein (HPr) are the general cytoplasmic PTS proteins involved in the transfer of most PTS carbohydrates in most bacteria. The substrate specificity of the PTS depends on the enzyme II complex, which consists of two soluble proteins/domains (EIIA and EIIB) and a membrane-bound protein/domain (EIIC). In a mannose-type PTS, the enzyme II complex also contains EIID, which is integrated into the membrane. EIIC and EIID facilitate the translocation of the substrate across the cell membrane. Bacteria contain several EII complexes: for example, *E. coli* contains at least 15 different ones. The phosphoryl transfer chain to phosphorylate the substrate (carbohydrate) begins with EI. PEP is the substrate of EI to provide a phosphoryl group that is then transferred to HPr. The phosphorylated HPr then phosphorylates one of the carbohydrate-specific EIIAs, which in turn passes the phosphoryl group to its cognate, EIIB. Finally, in most PTSs, the phosphorylated EIIB transfers the phosphoryl group to the carbohydrate bound to the cognate EIIC. In addition to sugar transport, the bacterial PTS has been reported to be involved in the regulation of carbohydrate metabolism (including carbohydrate catabolism repression and inducer exclusion), the utilization of a nitrogen source, and potassium uptake via protein-protein interactions. For example, EIIA^Glc^ (Crr, encoded by *crr* and formerly known as IIIglc or IIIGlc) interacts with MalK (the ATP-binding protein of the maltose/maltodextrin ABC transport system) and GlpK in *E. coli* and *Salmonella enterica* serovar Typhimurium, and Crr interacts with LacY (lactose permease) in *E. coli*. These interactions with Crr result in the inducer exclusion-mediated inhibition of MalK, GlpK and LacY and thereby prevent the uptake of maltose, glycerol and lactose, respectively (Deutscher et al., 2006, 2014). Wu et al. reported that in *K. pneumoniae*, the mutation of CelB, a cellobiose-specific EIIC, resulted in impaired biofilm formation (Wu et al., 2012). The peptide sequence identity between Crr in *K. pneumoniae* and *E. coli* is 98%. The mutation of *crr* in *K. pneumoniae* enhanced 1,3-PD production compared to the parent strain when bacteria were incubated in a mixture of glucose and glycerol under aerobic conditions (Oh et al., 2013).

Guo et al. (2010) reported that the non-capsuled *K. pneumoniae* provided a higher 1,3-PD yield. In our previous study, we found that the overexpression of *KPN00353-KPN00352*-*KPN00351* increased the capsular polysaccharide production in *K. pneumoniae*. *KPN00353* encodes 147-amino-acid (aa) putative EIIA homologue. *KPN00352* and *KPN00351* encode putative EIIB (95 aa) and EIIC (445 aa) homologues respectively (Horng et al., 2017). Therefore, the purpose of this study was to examine the effects of *KPN00353-KPN00352*-*KPN00351* on the 1,3-PD production. This study demonstrates the role of *KPN00353*, an open reading frame (ORF) common to several *K. pneumoniae* isolates from different sources, in binding to GlpK, glycerol uptake and 1,3-PD production under microaerobic conditions and provides structural insight into KPN00353-GlpK interaction.

## Materials and Methods

### Bacterial Strains and Growth Conditions

The bacterial strains and plasmids used in this study are listed in **Table 1**. The primers used in this study are listed in Supplementary Table S1. The bacteria were routinely cultured at 37°C in Luria-Bertani (LB) medium (10 g/L tryptone, 5 g/L yeast extract and 10 g/L NaCl) supplemented with the appropriate antibiotics. The analysis of 1,3-PD production by *K. pneumoniae* under microaerobic condition was performed by growing the bacteria in 250-mL and sealed flask containing 100 ml minimal culture media (MCM, 4.4 g/L K_2_HPO_4_, 1.3 g/L KH_2_PO_4_, 2 g/L (NH4)_2_SO_4_, 0.2 g/L MgSO_4_⋅7H_2_O, 1 g/L yeast extract, 20 g/L glycerol, 2 mL/L trace element solution and 1 mL/L Fe solution, pH 7.0) at 37°C. The trace element solution contained 70 mg/L ZnCl_2_, 100 mg/L MnCl_2_⋅4H_2_O, 60 mg/L H_3_BO_3_, 25 mg/L NiCl_2_⋅6H_2_O, 20 mg/L CoCl_2_⋅6H_2_O, 35 mg/L NaMoO_4_⋅H_2_O and 10.8 mM HCl. The Fe solution contained 5 g/L FeSO_4_⋅7H_2_O and 48 mM HCl. Microaerobic condition was kept by agitation without aeration (Chen X. et al., 2003).

**Table 1:** List of strains and plasmids used in the experiments.

Strain	Relevant genotype and phenotype	Reference or source
*E. coli* strains		
DH5α	F^-^, φ80d*lacZ*ΔM15, (*lacZYA-argF*) U169, *deoR, recA1, endA1, hsdR17*(rk^-^, m_k_^+^), *phoA, supE44, aaa*^-^, thi-1, *gyr*A96, *relA*1	Invitrogen
S17-1 aaapir	aaa-pir lysogen of CC118 [D(*ara-leu*) *araD* D*lacX*74 *galE galK phoA20 thi-1 rpsE rpoB argE*(Am) *recA1*]; permissive host for suicide plasmids requiring the Pir protein	Soo et al., 2005
BL21(DE3)pLysS	F^-^, *ompT, gal, dcm, lon, hsdS_B_*(r_B_^-^ m_B_^-^), *aaa*(DE3), pLysS(cm^R^)	Novagen
*K. pneumoniae* strains		
MGH78578	ATCC 700721	McClelland et al., 2000
KO353	MGH78578_ *KPN00353*::Sm, Sm^r^	This work
**Plasmid**		
pGEM-T easy	TA cloning vector, Ap^r^	Promega, Fitchburg, WI, United States
pGEX-1-SalI	GST tag expression vector constructed from pGEX-1 by inserting the restriction enzyme site *Sal*I between *EcoR*I and *Sma*I.	This work
pGEX-1::glpK::km	Expressing GST-tagged GlpK, Km^r^	This work
pET30b	Expression vector, His-tag, Km^r^	Novagen (merged with Merck-Millipore, Darmstadt, Germany)
pET30b::353WT	Expressing His-tagged KPN00353, Km^r^	This work
pET30b::H65Q	Expressing His-tagged mutated KPN00353(H65Q), Km^r^	This work
pET30b::H65D	Expressing His-tagged mutated KPN00353(H65D), Km^r^	This work
pET30b::H65E	Expressing His-tagged mutated KPN00353(H65E), Km^r^	This work
pET30b::H65R	Expressing His-tagged mutated KPN00353(H65R), Km^r^	This work
pET30b::H110Q	Expressing His-tagged mutated KPN00353(H110Q), Km^r^	This work
pET30b::mrkD	Expressing His-tagged MrkD, Km^r^	This work
pET30a::KPN00350	Expressing His-tagged KPN00350, Km^r^	This work
pET21::crr	Expressing His-tagged Crr (KPN02764), Amp^r^	This work
pBlueScript II SK+ (pBSK)	pBR322 ori, *lac* promoter, Amp^r^	Stratagene (La Jolla, CA, United States)
pBSK-Gm	Gentamycin resistance gene was inserted into the *Sca*I site within the ampicillin resistance gene in pBSK, Gm^r^	This work
pBSK-51-53-GM	pBSK::Gm containing *KPN00353- KPN00352-KPN00351* located downstream of *lac* promoter, Gm^r^	This work
pBSK-52-53-GM	pBSK::Gm containing *KPN00353-KPN00352*) located downstream of *lac* promoter, Gm^r^	This work
pBSK-53-GM	pBSK::Gm containing *KPN00353* located downstream of *lac* promoter, Gm^r^	This work
pBAD33	P_BAD_ promoter, pACYC184 ori, Cm^r^	Guzman et al., 1995
pBAD33::Histaq353WT	pBAD33 containing KPN00353, Cm^r^	This work
pBAD33::Histaq353H65Q	pBAD33 containing mutated KPN00353(H65Q), Cm^r^	This work
pBAD33::Histaq353H65D	pBAD33 containing mutated KPN00353(H65D), Cm^r^	This work
pBAD33::Histaq353H65E	pBAD33 containing mutated KPN00353(H65E), Cm^r^	This work
pBAD33::Histaq353H65R	pBAD33 containing mutated KPN00353(H65R), Cm^r^	This work
pBAD33::Histaq353H110Q	pBAD33 containing mutated KPN00353 (H110Q), Cm^r^	This work

### Reverse Transcription PCR (RT-PCR)

The bacterial RNA was extracted using the TRI reagent (Sigma–Aldrich, St. Louis, MO, United States). RNA (1.5 μg) was reverse transcribed using the Reverse Transcriptase Kit (Qiagen, Hilden, Germany) with random primers. Subsequently, the cDNA of each junction of *KPN00353-KPN00348* was amplified by PCR using cDNA (1 μL), primer pairs (400 nM; **Figure 2** and Supplementary Table S1), PCR buffer, dNTPs, and Taq polymerase according to the manufacturer’s instructions (Takara, Japan). The PCR conditions were 1 min at 95°C followed by 32 cycles of 15 s at 94°C, 30 s at 60°C, and 30 s at 72°C.

### Construction of *K. pneumoniae* Mutant Strain KO353

Genetic methods based on homologous recombination were used to amplify the central region of the *KPN00353* gene by PCR using the primer pair 353F/353R (Supplementary Table S1). The PCR products were TA-cloned into pGEM-T vectors (**Table 1**), excised as EcoRI fragments, and ligated with streptomycin-resistant Ω cassettes into *tnp*-deleted pUT vectors to form suicide plasmids (Soo et al., 2005). The suicide plasmids containing the central DNA region of *KPN00353* were transferred to *K. pneumoniae* MGH 78578 by electroporation. The transformants were spread on LB plates containing streptomycin (50 μg/mL). Mutant candidates were screened by colony PCR. PCR or Southern blot hybridization was performed to confirm the mutant genotypes.

### Analysis of 1,3-PD and Glycerol by High-Performance Liquid Chromatography (HPLC)

To quantify the 1,3-PD of *K. pneumoniae* MGH 78578, KO353 or bacteria containing the recombinant DNA under the control of the *lac* promoter, pre-cultures were grown overnight at 37°C in LB medium containing the appropriate antibiotics. Subsequently, the pre-cultures were diluted 100-fold and grown in MCM (supplemented with isopropyl β-D-1-thiogalactopyranoside (IPTG) at 1 mM for the bacteria containing the *lac* promoter) under microaerobic conditions for an additional 12 h at 37°C in a shaking incubator at 200 rpm without aeration before collection of the bacterial culture supernatant and quantification of 1,3-PD. To quantify the 1,3-PD in bacteria containing the recombinant DNA under the control of P_BAD_, the pre-cultures were diluted 100-fold in LB containing the appropriate antibiotics and grown for 2 h at 37°C under microaerobic conditions in a shaking incubator at 200 rpm without aeration. Then, arabinose (0.4%) was added to the medium for an additional 3 h at 30°C in a shaking incubator at 150 rpm. Then, the bacteria were changed to fresh MCM for an additional 4 h at 37°C before collection of the bacterial culture supernatant and quantification of 1,3-PD.

The bacterial growth was monitored by measuring the optical density of the broth culture at 600 nm, and then the bacterial culture supernatant was collected by centrifugation at 16,200 × *g* for 2 min and filtered using a nylon syringe-driven filter (Advangene, Lake Bluff, IL, United States). The concentrations of glycerol and 1,3-PD in the culture supernatant were determined using an HPLC system (Hitachi, Japan) equipped with an ICsep COREGEL-87H3 column (Transgenomic, San Jose, CA, United States) and a refractive index detector (L-2490). The column temperature was set to 65°C, and 5 mM H_2_SO_4_ was used as the mobile phase with a flow rate of 0.4 mL/min. The specific 1,3-PD production was defined as the amount of 1,3-PD per OD_600_ value of bacterial density.

### Construction of the Recombinant Plasmids pGEX-1::*glpk*::km and pET30b::353WT

Full-length *glpK* (*KPN04011*) and *KPN00353* were amplified by PCR using the chromosomal DNA of *K. pneumoniae* MGH 78578 and the primer pairs pGEX-glpK-FP/pGEX-glpK-RP and pET30b_353_FP/pET30b_353_RP, respectively (Supplementary Table S1). The PCR products of *glpK* and *KPN00353* were cloned into the pGEM-T easy vector (Promega, Fitchburg, WI, United States). Subsequently, the *glpK* fragment and the gene coding for kanamycin resistance were simultaneously cloned into the SmaI/SalI sites of pGEX-1-SalI (**Table 1**) to generate the pGEX-1::*glpK*::km plasmid for expressing glutathione S-transferase (GST)-tagged GlpK (*Kp*GlpK). The *KPN00353* DNA fragment was subcloned from the pGEM-T vector containing *KPN00353* into the SalI site of the pET30b vector (Novagen merged with Merck-Millipore Darmstadt, Germany) to generate the pET30b::353WT plasmid for expressing His-tagged KPN00353. The construction of the plasmids pET30b::mrkD, pET30a::KPN00350 and pET21::crr followed the same method used to construct pET30b::353WT with the appropriate primer pairs for the respective clones (Supplementary Table S1).

### Site-Directed Mutagenesis of KPN00353

The KPN00353 variants in pET30b::H65Q, pET30b::H65D, pET30b::H65E, pET30b::H65R and pET30b::H110Q were constructed by using the QuikChange^®^ Site-Directed Mutagenesis Kit (Stratagene, La Jolla, CA, United States) according to the manufacturer’s instructions. The primers are listed in Supplementary Table S1. The PCR conditions were as follows: 95°C for 5 min followed by 30 cycles of 95°C for 1 min, 50°C for 30 s, and 72°C for 6 min. The final step was an additional 72°C for 6 min. The template was pET30b::353WT (**Table 1**). The KPN00353 variants in pBAD33::Histaq353H65Q, pBAD33::Histaq353H65D, pBAD33::Histaq353H65E, pBAD33:: Histaq353H65R, and pBAD33::Histaq353H110Q were cloned from pET30b::H65Q, pET30b::H65D, pET30b::H65E, pET30b::H65R, and pET30b::H110Q, respectively, by XbaI and HindIII digestion followed by insertion into pBAD33 (Guzman et al., 1995).

### Protein Pull-Down Assay

The GST fusion and His-tagged fusion proteins were produced by culturing *E. coli* DH5α containing pGEX-1::*glpK*::km and *E. coli* BL21(DE3)pLysS containing pET30b::353WT (or pET30b carrying a His-tagged KPN00353 point-mutated gene), respectively, in 5 mL of LB medium containing antibiotics overnight. One hundred microliters of the overnight culture was transferred to 10 mL of LB broth containing antibiotics. After incubation at 37°C and 220 rpm for 2 h, IPTG was added at a final concentration of 0.5 mM for induction. After an additional 3 h of culture, the bacterial cells were pelleted by centrifugation (4000 × *g* for 10 min) and then suspended in 2 mL of STE buffer (10 mM Tris-HCl, pH 8.0, 150 mM NaCl, 1 mM EDTA, 1% Triton X-100, 1 mM PMSF, 1 μg/mL pepstatin, 1 μg/mL leupeptin and 1 mM DTT). The bacterial cells were frozen at -70°C overnight, lysed by sonication, and then centrifuged (4°C, 16,200 × *g*, 10 min). Two hundred microliters of supernatant containing GST-*Kp*GlpK and 800 μL of supernatant containing His-tagged proteins were mixed and incubated with mild shaking at 4°C for 1 h to allow the proteins to interact. Glutathione-sepharose (GSH) beads (50 μL; GE Healthcare, United Kingdom) were then added to the spent supernatant, and the mixture was incubated with mild shaking for 1 h at 4°C. After the beads were washed three times with PBS (pH 7.4), the proteins were separated by 12.5% sodium dodecyl sulfate-polyacrylamide gel electrophoresis (SDS-PAGE) and detected by Western blotting using anti-His antibody or anti-GST antibody.

### Western Blot Analysis

The Western blot procedures were modified from those described by Sambrook et al. (1989). The proteins were analyzed by 12.5% SDS-PAGE. The separated proteins were transferred to a nitrocellulose membrane (Pall, Washington, NY, United States) and incubated in blocking buffer (PBS with 5% milk and 0.1% Tween 20) for 1 h. Further incubation with anti-His monoclonal antibody (1:2500, Invitrogen) was performed in blocking buffer for 1 h at room temperature, and the membranes were then washed three times with PBS containing 0.1% Tween 20. After horseradish peroxidase-conjugated anti-mouse IgG antibody (1:2500, Invitrogen) was added, the membranes were incubated for 1 h, washed three times with PBS containing 0.1% Tween 20, and reacted with ECL Plus Solution (GE Healthcare, United Kingdom) for 1 min. The intensities of the bands were detected using the Gel Catcher 2850 chemiluminescence camera system (CLUBIO, Taipei, Taiwan).

### Quantification of Intracellular Glycerol 3-Phosphate (G3P)

*Klebsiella pneumoniae* containing pBAD33, pBAD33::HistaqKPN00353, pBAD33::Histaq353H65Q, pBAD33::Histaq353H65D, pBAD33::Histaq353H65E, pBAD33::Histaq353H65R, or pBAD33::Histaq353H110Q was grown overnight at 37°C in LB medium containing chloramphenicol (100 μg/mL). Then, the pre-cultures were diluted 100-fold in fresh LB medium containing chloramphenicol (100 μg/mL). After the cultures were grown at 37°C in a shaking incubator at 200 rpm for 2 h, 0.4% arabinose was added for protein induction. After induction at 30°C and 150 rpm for 3 h, the bacteria were centrifuged and resuspended in fresh MCM. After 4 h of growth, the cell density was measured by spectrophotometry at 600 nm. An aliquot [OD × V(ml) = 1] of cells was centrifuged and homogenized in 400 μL of G3P assay buffer provided in the Glycerol 3-Phosphate Colorimetric Assay Kit (Sigma–Aldrich, St Louis, MO, United States). The intracellular G3P was then measured according to the manufacturer’s instructions using the Glycerol 3-Phosphate Colorimetric Assay Kit (Sigma–Aldrich, St Louis, MO, United States).

### Structural Modeling

To predict the interface residues of the *K. pneumoniae Kp*GlpK-KPN00353 complex from a structural perspective, 3D structural models of *K. pneumoniae* GlpK (*Kp*GlpK) and KPN00353 were created using the HHpred server (Soding et al., 2005), a website that uses HMM-HMM comparisons to detect homology and predict structures. The structural model of *Kp*GlpK was generated using the top 10 templates with the highest scores (PDB accession codes: 3H3N_X, 3G25_A, 3EZW_A, 2D4W_A, 2DPN_A, 2ZF5_O, 2W40_A, 4ELJ_A, 3LL3_A, and 3WXL_A). The structural model of KPN00353 was generated using seven templates selected from the nine templates with highest scores (PDB accession codes: 3OXP_A, 1A3A_A, 1A6J_A, 2A0J_A, 3BJV_A, 3URR_A, and 2OQT_A). The structural model of *K. pneumoniae Kp*GlpK-KPN00353 complex was generated using the ZDOCK server (Chen R. et al., 2003), a website that performs a full rigid-body search of docking orientations between two proteins. The top docking model of the *Kp*GlpK-KPN00353 complex was superimposed with the *E. coli* GlpK-Crr complex structure (PDB accession code: 1GLA) using the PDBeFold server (Krissinel and Henrick, 2004) to produce structural figures. The models of the KPN00353 mutants were generated by the Coot software (Emsley and Cowtan, 2004). The interface residues of all complex structures were determined by PDBePISA (Krissinel and Henrick, 2007), a website that provides an interactive tool for exploring macromolecular interfaces. Structural figures were then produced using PyMOL (DeLano Scientific^1^).

### Statistical Methods

For all quantitative data, the values were expressed as mean ± standard deviation from three independent bacterial cultures. Paired Student’s *t*-test was performed to determine statistically significant differences, and *p* < 0.05 was considered to indicate statistical significance. For RT-PCR, Western blotting and SDS-PAGE, the representative data were chosen from three independent experiments.

## Results

### The Locus of *KPN00353* and Its Downstream Region Is Common to Most *K. pneumoniae* Isolates

**FIGURE 1:**
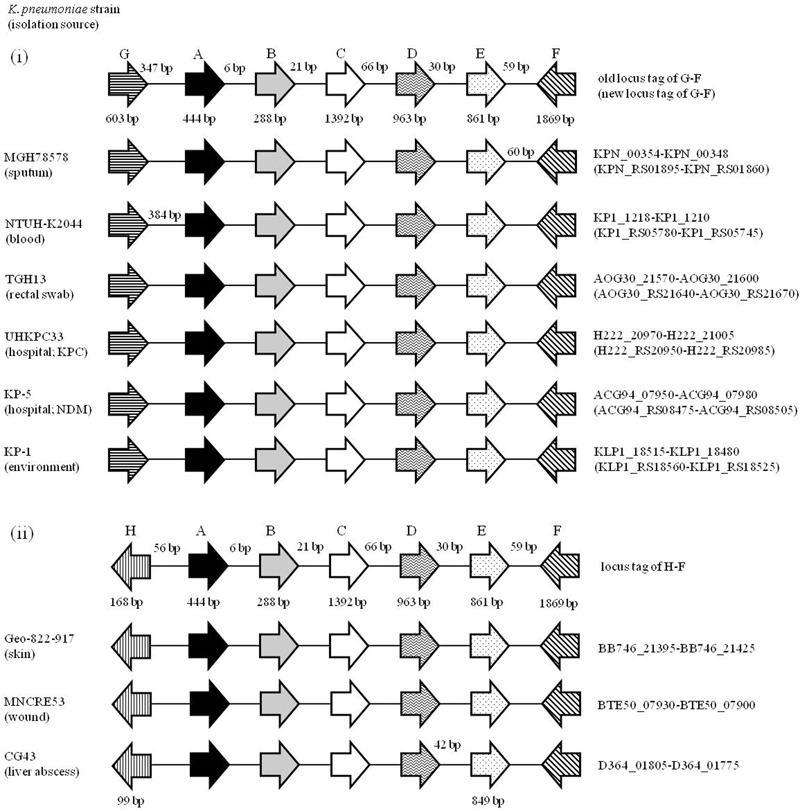
Alignment of the *KPN00354-KPN00348* locus in different *K. pneumoniae* isolates. Open reading frames (ORFs) A, B, C, D, E, F and G are homologues of KPN00353, KPN00352, KPN00351, KPN00350, KPN00349, KPN00348 and KPN00354, respectively. ORF A is an EIIA homologue (black arrow). ORF B is an EIIB homologue (gray arrow). ORF C is an EIIC homologue (white arrow). ORF D is a protein containing the PfkB-specific motif (arrow with wavy line inside). ORF E is a putative type II Fba (arrow with dot inside). ORF F is predicted to encode amidohydrolase (arrow with slash inside). ORF G is predicted to encode peroxiredoxin (arrow with horizontal line). ORF H is a hypothetical protein (arrow with vertical line). Arrows denote the direction of transcription. The gene lengths and the lengths between each ORF are shown in representative graphs but the lengths different from representative graphs are shown on separate graphs.

To see whether the ORFs *KPN00353-KPN00352*-*KPN00351* are specific to *K. pneumoniae* MGH 78578 or common to most *K. pneumoniae* isolates, we randomly selected eight *K. pneumoniae* strains from the NCBI database and compared their genomes with that of *K. pneumoniae* MGH 78578. These nine *K. pneumoniae* isolates were isolated from different sources including sputum, rectal swab, blood, liver abscess, skin, wound and environmental samples. Among them, one NDM-positive strain and one KPC-positive strain were chosen to test whether isolates with these drug-resistant phenotypes have the locus *KPN00353-KPN00352*-*KPN00351* (**Figure 1**). The results showed that the locus from KPN00353 to its 6-kb downstream region can be observed in all *K. pneumoniae* isolates. However, these nine isolates can be separated into two groups according to the upstream region of KPN00353. One group consists of isolates with a peroxiredoxin (KPN00354) located 347 bp upstream of KPN00353 in most isolates or 384 bp upstream of the KPN00353 homologue in NTU-K2044. The other group consists of isolates with an ORF of unknown function located 56 bp upstream of the KPN00353 homologue. This 6-kb locus cannot be found in the genome of *Klebsiella oxytoca*. Therefore, we presume that the observation in this study of *KPN00353* in *K. pneumoniae* MGH 78578 is common to most *K. pneumoniae* isolates.

**FIGURE 2:**
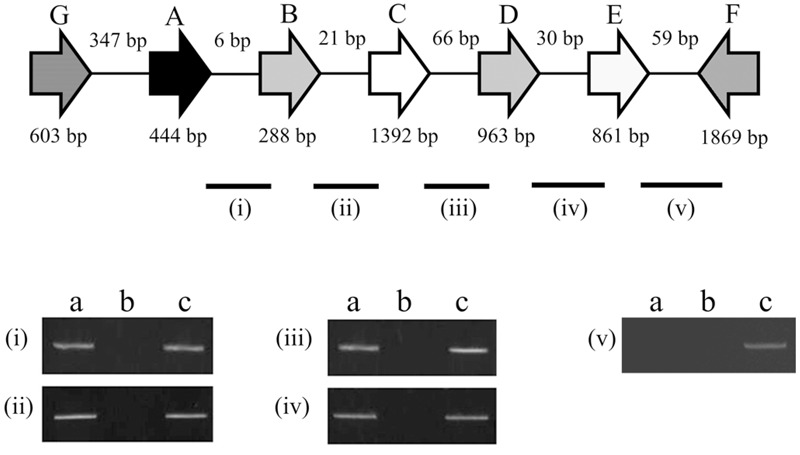
Schematic diagram of the genetic organization of *KPN00353* (A), *KPN00352* (B), *KPN00351* (C), *KPN00350* (D), *KPN00349* (E), *KPN00348* (F) and *KPN00354* (G) in *K. pneumoniae* MGH 78578. Arrows denote the direction of transcription. Each line indicates a junction (i, ii, iii, iv, and v) between the ORFs. The RNA extracted from wild-type *K. pneumoniae* was analyzed by RT-PCR (a) or PCR (b). The genomic DNA of the wild-type strain was analyzed by PCR as a positive control (c).

The peptide sequence from the 23rd to the 299th residue of KPN00350 contains the PfkB-specific motif. The gene *KPN00349* encodes a 286-amino-acid product that is a putative type II Fba. The distances between *KPN00353* and *KPN00352, KPN00352* and *KPN00351, KPN00351* and *KPN00350*, and *KPN00350* and *KPN00349* were found to be 5, 74, 65 and 29 bp, respectively. Therefore, we predicted these five genes to be in an operon. To determine the transcriptional unit containing these five genes, reverse transcription PCR (RT-PCR) was performed using the total RNA extracted from *K. pneumoniae* MGH 78578 and primers that specifically amplify the regions between each pair of ORFs (junctions i, ii, iii and iv in **Figure 2**). The transcripts were detected with each primer pair, demonstrating that *KPN00353, KPN00352, KPN00351, KPN00350* and *KPN00349* are transcribed into a polycistronic mRNA (**Figure 2**). *KPN00348* is located downstream and in the opposite direction of *KPN00349*. *KPN00348* is predicted to encode amidohydrolase (**Figures 1, 2**). The region between *KPN00349* and *KPN00348* in the extracted RNA, as a negative control, could not be amplified by RT-PCR using the corresponding primers (junction v in **Figure 2**). Therefore, *KPN00353, KPN00352, KPN00351, KPN00350* and *KPN00349* are in an operon.

### Overexpression of KPN00353 Represses 1,3-PD Production in *K. pneumoniae*

**FIGURE 3:**
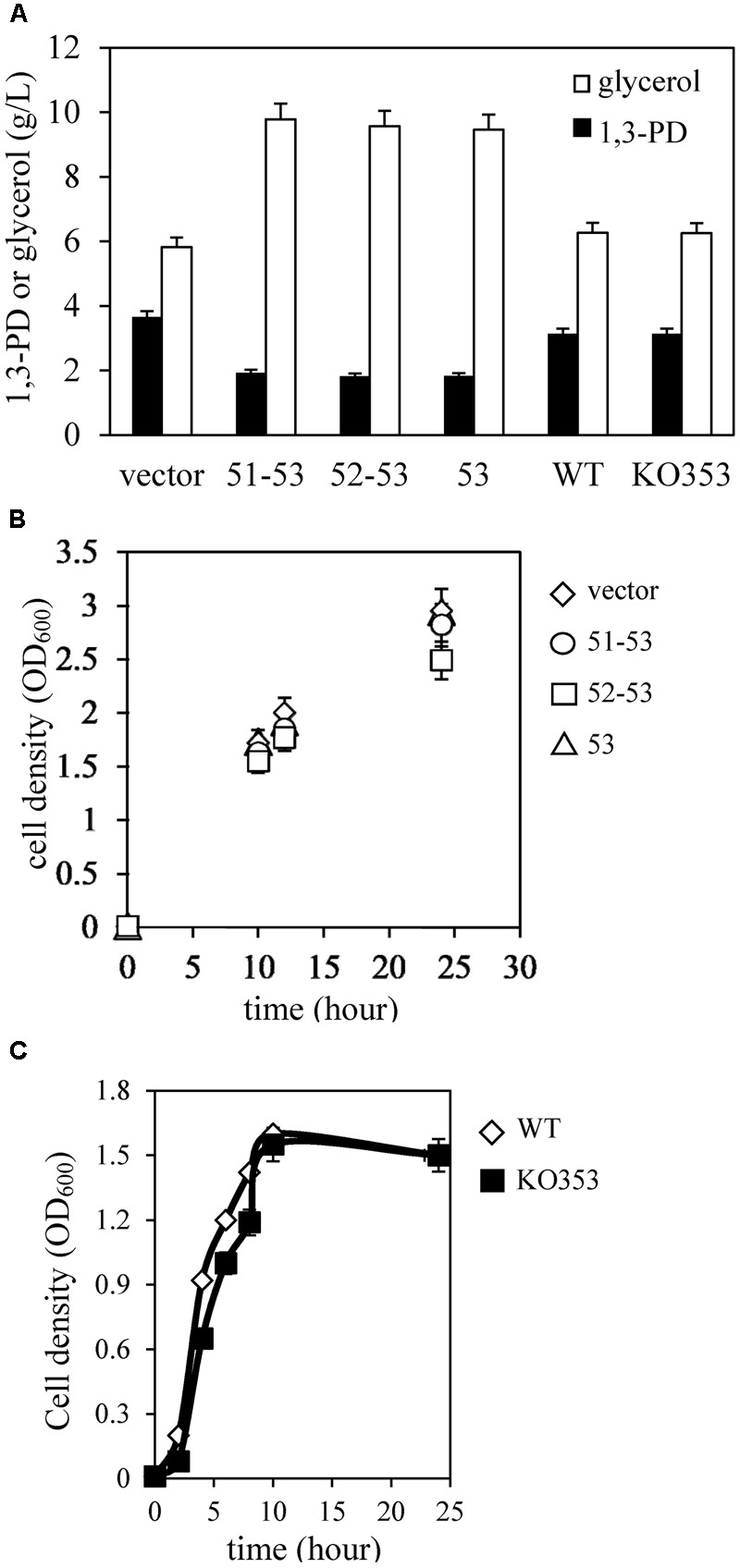
Quantification of 1,3-PD, glycerol and cell density. **(A)** The amount of 1,3-PD (black bar) and residual glycerol (white bar) in the medium after 12 h of bacterial culture. **(B)** The bacterial density was measured at OD_600_ when the bacteria were inoculated and after growing for 10, 12, and 24 h. vector, *K. pneumoniae* containing pBSK-Gm as vector control (white diamond). 51–53, *K. pneumoniae* containing the plasmid pBSK-51–53-GM to overexpress KPN00353, KPN00352 and KPN00351 (white circle). 52–53, *K. pneumoniae* containing the plasmid pBSK-52–53-GM to overexpress KPN00353 and KPN00352 (white square). 53, *K. pneumoniae* containing the plasmid pBSK-53-GM to overexpress KPN00353 (white triangle). **(C)** The bacterial density was measured at OD_600_. White diamond, wild type (WT). Black square, KPN00353 mutant (KO353).

We observed reduced 1,3-PD production in this study (**Figure 3A**) and increased capsular polysaccharide production in the previous study (Horng et al., 2017) in *K. pneumoniae* overexpressing *KPN00353-KPN00352*-*KPN00351* incubated under microaerobic conditions compared with that of the vector control. This finding is consistent with the study by Guo et al. (2010) reporting that non-capsulated *K. pneumoniae* provided a higher 1,3-PD yield. Then, we tried to detect which ORF(s) of *KPN00353-KPN00352*-*KPN00351* affected the 1,3-PD production by constructing plasmids controlling the expression of KPN00352-KPN00353 or KPN00353 by an inducible *lac* promoter and then transforming these plasmids separately into *K. pneumoniae* MGH 78578. The results showed that reductions in the level of 1,3-PD production were similar in bacteria overexpressing *KPN00353-KPN00352*-*KPN00351, KPN00353-KPN00352* or *KPN00353* but not in the vector control (**Figure 3A**). Therefore, we conclude that KPN00353 affects 1,3-PD production in *K. pneumoniae*. Subsequently, a *KPN00353* mutant KO353 was constructed. The 1,3-PD production and growth in the KO353 and wild-type were not significantly different, indicating that the deficiency of *KPN00353* did not affect the 1,3-PD production in *K. pneumoniae* (**Figures 3A,C**). Since 1,3-PD is produced from glycerol fermentation in *K. pneumoniae*, we observed the residual glycerol in the medium after a 12-h bacterial culture under microaerobic conditions. HPLC analysis showed that 9.46 g/L glycerol remained in the medium after culturing *K. pneumoniae* overexpressing KPN00353 (**Figure 3A**). However, 5.82 g/L glycerol remained in the medium after incubating *K. pneumoniae* containing the vector control (**Figure 3A**). To determine whether the growth was affected by the glycerol uptake, we examined the bacterial growth, and the results showed no significant difference between *K. pneumoniae* containing the vector control or overexpressing KPN00353 (**Figure 3B**). These results indicated that overexpression of KPN00353 reduced 1,3-PD production and glycerol uptake by *K. pneumoniae* without affecting bacterial growth in a medium containing glycerol and yeast extract as the carbon sources under microaerobic conditions.

### The Interaction of KPN00353 and GlpK Inhibits GlpK Activity

**FIGURE 4:**
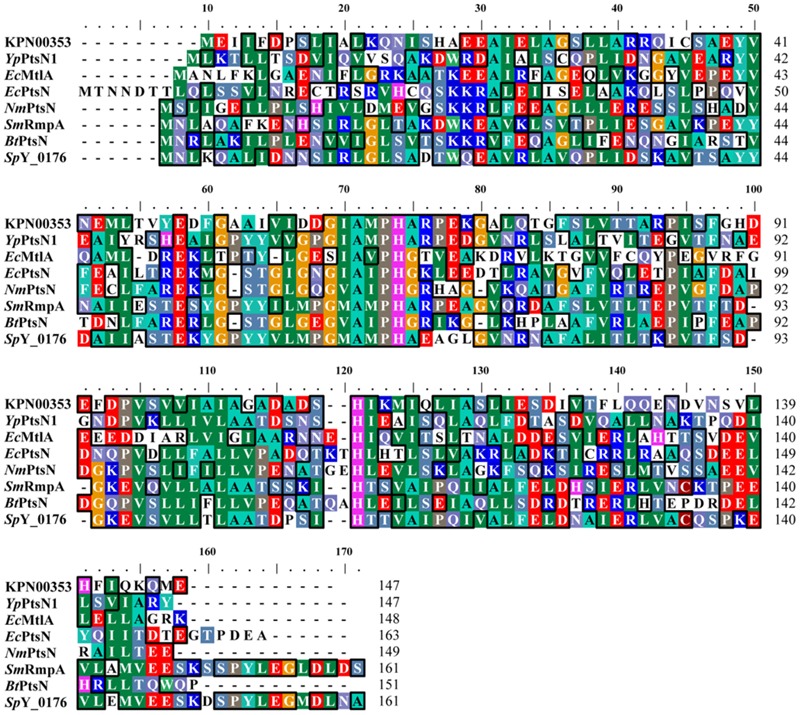
Multiple sequence alignment of PTS-fructose-mannitol EIIA family proteins. Multiple alignment was performed using ClustalW (Thompson et al., 1994; Chenna et al., 2003). *Yp*PtsN1: *Yersinia pestis* EIIA^Mtl^ (PDB accession code: 3OXP_A); *Ec*MtlA: *E. coli* EIIA^Mtl^ (PDB accession code: 1A3A_A); *Ec*PtsN: *E. coli* EIIA^Ntr^ (PDB accession code: 1A6J_A); *Nm*PtsN: *Neisseria meningitides* EIIA^Ntr^ (PDB accession code: 2A0J_A); *Sm*RmpA: *Streptococcus mutans* PTS IIA fructose superfamily (PDB accession code: 3BJV_A); *Bt*PtsN: *Burkholderia thailandensis* EIIA^Ntr^ (PDB accession code: 3URR_A); and *Sp*Y_0176: *Streptococcus pyogenes* PTS IIA fructose superfamily (PDB accession code: 2OQT_A).

**FIGURE 5:**
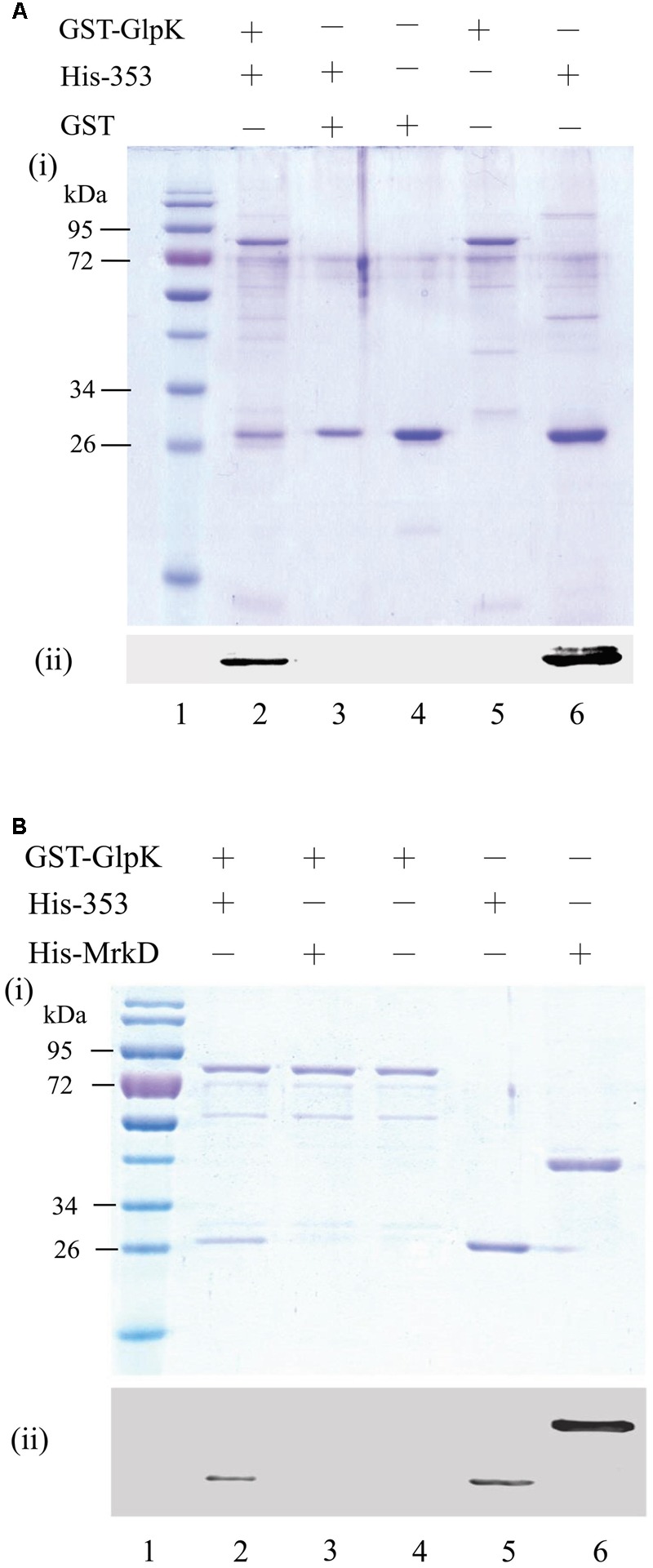
Interaction between KPN00353 and GlpK. GST-tagged GlpK (GST-GlpK) was allowed to interact with His-tagged protein (lane 2, 3 in **A,B**), and then the proteins captured on GSH-Sepharose beads were analyzed by (i) SDS-PAGE followed by Coomassie Blue staining and (ii) Western blotting using anti-His antibody. The purified proteins were analyzed simultaneously with the control (lane 4, 5, 6 in **A,B**). The protein marker is shown in lane 1. His-353, His-tagged KPN00353; His-MrkD, His-tagged MrkD; GST, glutathione S-transferase.

Iterative database searches performed using the protein BLAST (BLASTP) suite demonstrated that the primary sequence of KPN00353 was similar to that of the fructose-mannitol EIIA (EII^Fru^ and EIIA^Mtl^) family, a subfamily of the glucose-fructose-lactose PTS superfamily (**Figure 4**). Crr is a glucose-specific EIIA (EIIA^Glc^) and is classified as part of the glucose family, another subfamily of the glucose-fructose-lactose PTS superfamily. Unphosphorylated Crr protein in *E. coli* and *S. enterica* serovar Typhimurium was reported to interact with GlpK, leading to the inhibition of glycerol uptake (Deutscher et al., 2006, 2014). The primary peptide sequences of Crr and KPN00353 are not similar and are classified into two different subfamilies. However, we still attempted to test whether KPN00353 interacted with KPN04011, the GlpK homologue in *K. pneumoniae* MGH 78578. *KPN04011* was constructed downstream of the GST gene in the plasmid pGEX-1::glpK::km to generate GST-tagged *Kp*GlpK (**Table 1**). The plasmid pET30b::KPN00353 was constructed to express His-tagged KPN00353 (**Table 1**). In addition, His-tagged MrkD, the type 3 fimbrial adhesin (Schurtz et al., 1994), was purified and used as a negative binding control. The GST-*Kp*GlpK, GST and His-tagged proteins were expressed in *E. coli*. GST-*Kp*GlpK or GST was immobilized on GSH-Sepharose beads. His-tagged KPN00353 was pulled down by GST-*Kp*GlpK but not by GST alone (**Figure 5A**). Furthermore, His-tagged MrkD was not pulled down by GST-*Kp*GlpK (**Figure 5B**). These results indicated that KPN00353 interacted specifically with *Kp*GlpK.

**FIGURE 6:**
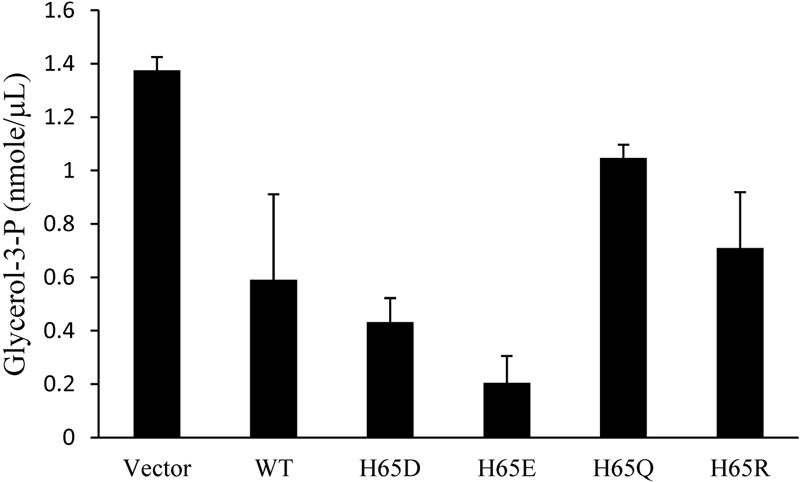
Effects of KPN00353 overexpression on intracellular glycerol-3-phosphate concentration. We quantified the concentration of glycerol-3-phosphate (glycerol-3-P) in *K. pneumoniae* containing pBAD33 (Vector), *K. pneumoniae* containing pBAD33::Histaq353WT (WT), *K. pneumoniae* containing pBAD33::Histaq353H65D (H65D), *K. pneumoniae* containing pBAD33::Histaq353H65E (H65E), and *K. pneumoniae* containing pBAD33::Histaq353H65Q (H65Q) or pBAD33::Histaq353H65R (H65R).

Glycerol is phosphorylated to G3P by GlpK (Wehtje et al., 1995). To examine the effect of the binding of KPN00353 and GlpK on the activity of GlpK, we quantified the intracellular G3P in recombinant *K. pneumoniae* either overexpressing KPN00353 or containing the vector, respectively. Compared to the vector control, *K. pneumoniae* overexpressing wild-type KPN00353 decreased the intracellular G3P concentration (**Figure 6**). This decrease indicated that the interaction of KPN00353 and GlpK inhibited the activity of GlpK in *K. pneumoniae*.

### Structural Models of KPN00353 Bound to GlpK of *K. pneumoniae*

**FIGURE 7:**
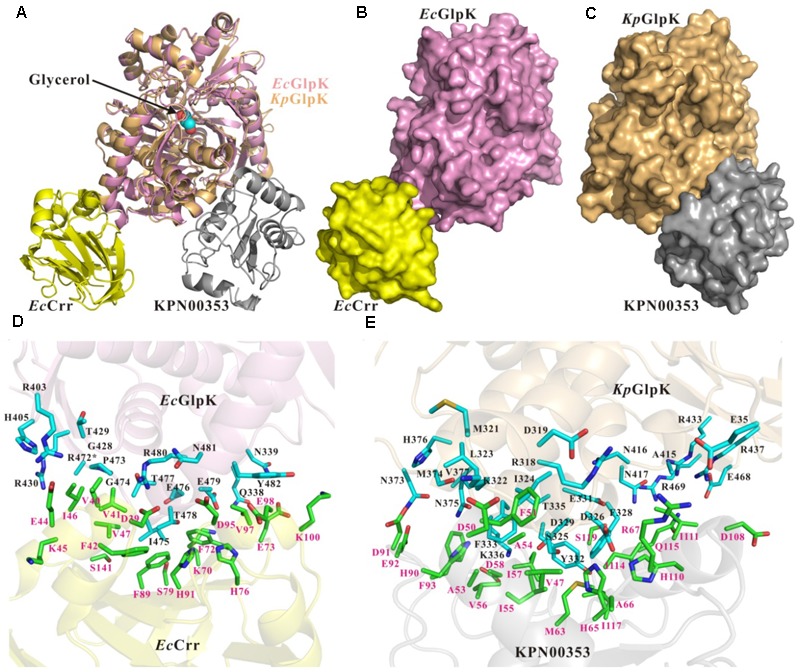
Structures and interface residues of the *E. coli* GlpK-Crr complex and *K. pneumoniae* GlpK-KPN00353 complex. **(A)** Superposition of cartoon representations of *E. coli* GlpK-Crr complex (solved structure, PDB accession code 1GLA) and *K. pneumoniae* GlpK-KPN00353 complex (modeled structure). Surface representations of **(B)** the *E. coli* GlpK-Crr complex and **(C)** the *K. pneumoniae* GlpK-KPN00353 complex in the same orientation as in **(A)**. **(D,E)** Close-up view of the interfaces of the *E. coli* GlpK-Crr complex and the *K. pneumoniae* GlpK-KPN00353 complex, respectively. The *E. coli* GlpK, *E. coli* Crr, *K. pneumoniae* GlpK and KPN00353 molecules are shown in pink, yellow, light-orange and gray, respectively. The side chains of the interface residues of GlpK molecules are shown in cyan (carbon atoms) stick models and labeled in black. The side chains of the interface residues of the Crr and KPN00353 molecules are shown in green (carbon atoms) stick models and labeled in magenta. The glycerol molecule is shown in a spheres model. Oxygen atoms are shown in red and nitrogen atoms in blue. ^∗^The R472 side-chain atoms of *E. coli* GlpK are missing atoms in the 1GLA coordination file.

The primary sequence of KPN00353 is similar to that of the fructose-mannitol EIIA (EII^Fru^ and EIIA^Mtl^) family, according to the sequence analysis by BLASTP. Owing to the absence of structural information on the fructose-mannitol family proteins interacting with GlpK, we generated individual structural models of *Kp*GlpK and KPN00353 using the HHpred server and a complex structural model of *Kp*GlpK interacting with KPN00353 using the ZDOCK server, which provided a structural perspective for understanding the interactions between *Kp*GlpK and KPN00353 (**Figure 7** and **Table 2**). Our *Kp*GlpK-KPN00353 complex model revealed that KPN00353 and *Kp*Crr might use individual binding sites to interact with *Kp*GlpK (**Figure 7**). Comparing the protein structures of EII^Fru^/EIIA^Mtl^ to EIIA^Glc^ and EIIA^Lac^ showed that the position of His65 in KPN00353 was similar to that of His91 of *Ec*Crr (EIIA^Glc^ in *E. coli*) (**Figures 7D,E**). Comparing the predicted KPN00353 structure and the solved EIIA^Fru^ structures showed that another key conserved residue in KPN00353 is His110, which is proximal to the His65 of KPN00353 residues (**Figure 7E**). The structural position of His110 in KPN00353 is similar to that of His76 in *Ec*Crr (**Figures 7D,E**) (Hurley et al., 1993; Sliz et al., 1997; Bordo et al., 1998). Both key conserved residues involved in the phosphorylation regulation of KPN00353, His65 and His110, are located at the center of the interface of *Kp*GlpK-KPN00353 complex (**Figure 7E**). The interface residues of the *Ec*GlpK-Crr complex (solved structure) and the *Kp*GlpK-KPN00353 complex (modeled structure) are listed in **Table 2**.

**Table 2:** The interface residues of the *E. coli* GlpK-Crr complex (solved structure, PDB accession code 1GLA) and the *K. pneumoniae* GlpK-KPN00353 complex (modeled structure).

Interface residues of*Ec*GlpK-Crr complex (PDB accession code 1GLA)		Interface residues of *Kp*GlpK-KPN00353 complex (modeled structure)	
***Ec*GlpK**	***Ec*Crr**	***Kp*GlpK**	**KPN00353**

Q338	D39	E35	V47
N339	V40	R318	D50
R403	V41	D319	F51
H405	F42	M321	G52
G428	E44	K322	A53
T429	K45	L323	A54
R430	I46	I324	I55
R472	V47	S325	V56
P473	K70	D326	I57
G474	F72	F328	D58
I475	E73	D329	M63
E476	H76^b^	E331	**H65^a^**
T477	S79	Y332	A66
T478	F89	F333	R67
E479	**H91^a^**	T335	H90
R480	D95	K336	D91
N481	V97	V337	E92
Y482	E98	N373	F93
	K100	S374	D108
	S141	N375	H110^b^
		H376	I111
		A415	I114
		N416	Q115
		N417	I117
		R433	A118
		R437	S119
		E468	
		R469	

*The interface residues were determined by PDBePISA, a website providing an interactive tool for the exploration of macromolecular interfaces. ^a^The histidine residue receiving the phosphoryl group from the donor HPr is indicated in bold. ^b^The histidine residue closing the phosphorylation site is underlined.*

### Residue His65 of KPN00353 Is Important for Binding to GlpK and 1,3-PD Production

**FIGURE 8:**
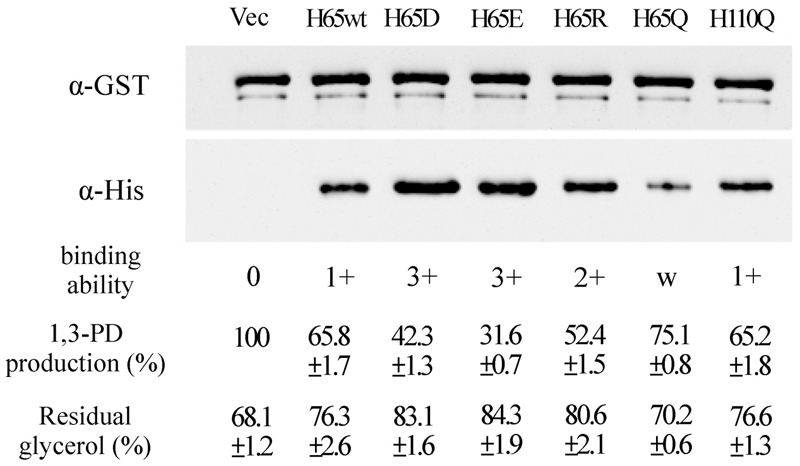
Effects of the interaction between GlpK and the KPN00353 variants on 1,3-PD production. After the interaction of GST-tagged GlpK with wild-type or variant His-tagged KPN00353, the proteins captured on GSH-Sepharose beads were analyzed by Western blotting using either anti-GST antibody (α-GST) or anti-His antibody (α-His). From the Western blotting results, we defined the binding affinity of GlpK and wild-type KPN00353 (WT) to be 1+. The binding affinity of GlpK and the KPN00353 variant was comparable to that of GlpK and H65wt. H65wt, *E. coli* with pET30b::353WT; H65D, *E. coli* with pET30b::H65D; H65E, *E. coli* with pET30b::H65E; H65R, *E. coli* with pET30b::H65R; H65Q, *E. coli* with pET30b::H65Q; H110Q, *E. coli* with pET30b::H110Q. The 1,3-PD production of *K. pneumoniae* with pBAD33 (Vec) after incubation for 4 h was 0.186 g/L as 100%. The 1,3-PD production of *K. pneumoniae* with plasmids expressing wild-type or variant KPN00353 was compared to that of Vec. The residual glycerol in the medium was compared to the glycerol concentration in fresh MCM. H65wt, *K. pneumoniae* with pBAD33::Histaq353WT; H65D, *K. pneumoniae* with pBAD33::Histaq353H65D; H65E, *K. pneumoniae* with pBAD33::Histaq353H65E; H65R, *K. pneumoniae* with pBAD33::Histaq353H65R; H65Q, *K. pneumoniae* with pBAD33::Histaq353H65Q; H110Q, *K. pneumoniae* with pBAD33::Histaq353H110Q.

To study the roles of residues His65 and His110 of KPN00353 in the interaction with GlpK, we generated His-tagged KPN00353 variants in which histidine was replaced with glutamine (H65Q and H110Q), aspartate (H65D), glutamate (H65E) or arginine (H65R). In the protein pull-down assay, GST-tagged GlpK bound to His-tagged H65D or His-tagged H65E mutant proteins more strongly than to His-tagged H65R or to His-tagged wild-type KPN00353 and bound weakly to His-tagged H65Q (**Figure 8**). The binding affinity between GlpK and the His-tagged H110Q mutant protein was similar to that between GlpK and His-tagged wild-type KPN00353 (**Figure 8**). To examine whether the binding affinity of KPN00353 variant and GlpK correlated with 1,3-PD production, we constructed plasmids containing the mutated KPN00353 genes under the control of the pBAD promoter and transformed them into *K. pneumoniae* MGH 78578 (**Table 1**). Then, we quantified the 1,3-PD from the recombinant *K. pneumoniae* overexpressing the KPN00353 variant. Compared with the vector control, all strains overexpressing the KPN00353 wild-type or variant protein exhibited reduced 1,3-PD production (**Figure 8**). Among them, *K. pneumoniae* overexpressing the H65D, H65E or H65R variant produced lower levels of 1,3-PD than *K. pneumoniae* overexpressing the wild-type KPN00353. The 1,3-PD production of the strain overexpressing the H65Q variant was higher than that of *K. pneumoniae* overexpressing wild-type KPN00353 (**Figure 8**). The strain with the overexpressed H110Q variant produced a similar amount of 1,3-PD to that of the strain overexpressing wild-type KPN00353 (**Figure 8**). Furthermore, observation of the residual glycerol in the medium growing these bacteria showed an inverse relationship with 1,3-PD production: the lower the 1,3-PD production, the higher was the residual glycerol in the medium (**Figure 8**). Moreover, to examine the effect of the overexpression of different KPN00353 variants on the activity of GlpK, we quantified the intracellular G3P in recombinant *K. pneumoniae* overexpressing wild-type or variant KPN00353 proteins. The results of the G3P colorimetric assay showed that the amount of G3P was reduced in the strains overexpressing wild-type or variant KPN00353 proteins compared with that in the vector control (**Figure 6**). Among them, *K. pneumoniae* overexpressing the H65E variant produced the lowest level of G3P, while *K. pneumoniae* overexpressing H65Q produced a lesser reduction in G3P level than *K. pneumoniae* overexpressing wild-type KPN00353 (**Figure 6**). We did not test the G3P in *K. pneumoniae* overexpressing H110Q variant because the binding affinity of GlpK and the H110Q variant and the 1,3-PD production of recombinant *K. pneumoniae* overexpressing the H110Q variant were not significantly different from the corresponding values for the wild-type KPN00353 (**Figure 8**). Therefore, these results indicated that the overexpression of KPN00353 inhibited the function of GlpK by binding to GlpK, leading to decreased 1,3-PD production.

### Structure Modeling and Comparison of Wild-Type and Mutant KPN00353

**FIGURE 9:**
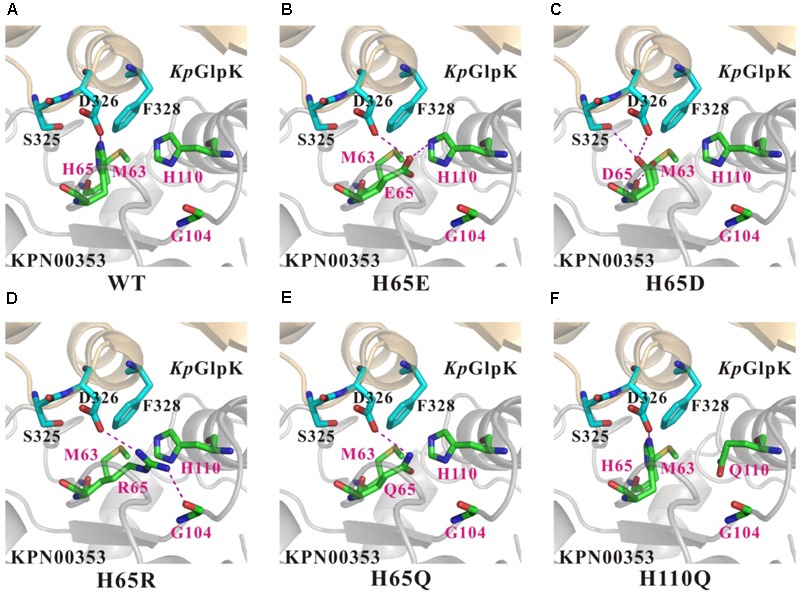
Comparison of the contacts surrounding residues 65 and 110 of KPN00353 variants interacting with *K. pneumoniae* GlpK. **(A)** Wild-type KPN00353, **(B)** KPN00353 H65E mutant, **(C)** KPN00353 H65D mutant, **(D)** KPN00353 H65R mutant, **(E)** KPN00353 H65Q mutant, and **(F)** KPN00353 H110Q mutant interacting with *K. pneumoniae* GlpK. The main chain and side chains of the interface residues of the GlpK molecules are shown in cyan (carbon atoms) stick models and labeled in black. The main chains and side chains of the interface residues of the KPN00353 molecules are shown in green (carbon atoms) stick models and labeled in magenta. The critical salt bridges or hydrogen bonds are indicated by magenta dashed lines.

To better understand the effects of the KPN00353 mutants on the binding affinity of *Kp*GlpK-KPN00353 complex formation, structural models of *Kp*GlpK complexed with the KPN00353 mutants were generated to interpret the contributions of the His65 and His110 residues of KPN00353 to the interface of the *Kp*GlpK-KNP00353 complex. Residue His65 of KPN00353 may form a salt bridge with residue Asp326 of *Kp*GlpK at physiological pH (**Figure 9A**). Moreover, residues His65 and His110 of KPN00353 can both contribute hydrophobic interactions with residue Phe328 of *Kp*GlpK (**Figure 9A**). The H65E mutant of KPN00353 may form a hydrogen bond with residue Asp326 of *Kp*GlpK and a salt bridge with residue His110 of KPN00353 at physiological pH (**Figure 9B**). The H65D mutant of KPN00353 may form hydrogen bonds with residues Ser325 and Asp326 of *Kp*GlpK and the main chain of residue Met63 of KPN00353 at physiological pH (**Figure 9C**). The H65R mutant of KPN00353 may form a salt bridge with residue Asp326 of *Kp*GlpK and a hydrogen bond with the main chain of residue Gly104 of KPN00353 at physiological pH (**Figure 9D**). The H65Q mutant of KPN00353 may form a hydrogen bond with residue Asp326 of *Kp*GlpK at physiological pH (**Figure 9E**). The H65E, H65D, H65R, H65Q and H110Q mutants of KPN00353 all reduce the hydrophobic interactions with residue Phe328 of *Kp*GlpK (**Figure 9**).

## Discussion

In this study, we report that the overexpression of KPN00353, an EIIA homologue in *K. pneumoniae*, reduced both 1,3-PD production and glycerol uptake under microaerobic conditions via directly inhibiting the activity of GlpK. The KPN00353 homologue can be found in most *K*. *pneumoniae* isolates (**Figure 1**). Therefore, our findings regarding KPN00353 in *K. pneumoniae* MGH78578 can be applied to other *K. pneumoniae* isolates. However, we do not know what kind of environmental signal led to the inhibition of GlpK by KPN00353 in *K. pneumoniae* in this study. In *E. coli* and *S. enterica* serovar Typhimurium, environmental glucose decreases the extent of phosphorylation of EIIA^Glc^, while unphosphorylated EIIA^Glc^ binds efficiently to GlpK and inhibits the activity of GlpK in the presence of both glycerol and glucose (Postma et al., 1984; Deutscher et al., 2006). The overexpression of EIIA^Glc^ tends to be in the unphosphorylated form (Eppler et al., 2002). Therefore, in this study, we suppose that most overexpressed KPN00353 is unphosphorylated type and apt to bind with GlpK. Because we have not found the cognate sugar of KPN00353, overexpression of KPN00353 in medium containing glycerol without sugar may simulate the unphosphorylated state of KPN00353 in medium containing glycerol with the cognate sugar under microaerobic conditions. We presumed that the cognate sugar of KPN00353 has priority over glycerol for catabolism in *K. pneumoniae* under microaerobic conditions, leading reduced 1,3-PD production when the cognate sugar of KPN00353 and glycerol are both present in the medium.

**FIGURE 10:**
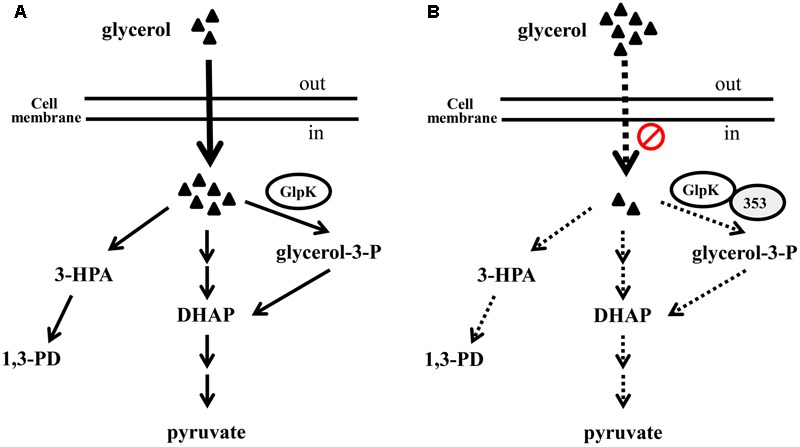
The hypothetical role of KPN00353 in glycerol metabolism in *K. pneumoniae*. Glycerol is metabolized by respiratory metabolism which GlpK is involved in and oxidative fermentation to produced DHAP or by reductive pathway to produce 1,3-PD under (micro)aerobic condition **(A)**. We hypothesize that the interaction of GlpK and KPN00353 (353) decreases the glycerol uptake into bacterium (in **B**). The reduction of intracellular glycerol results in the low activity of glycerol metabolism. Dash lines in (B) indicate the activities of reactions or pathways in **(B)** are lower than those represented by solid lines in **(A)**. Triangle indicates glycerol.

The 1,3-PD production of the *K. pneumoniae glpK* mutant and of the parent strain under anaerobic conditions did not differ significantly (Ashok et al., 2013). However, in our study, GlpK inhibition was correlated with reductions in G3P synthesis, glycerol uptake and 1,3-PD production in *K. pneumoniae* under microaerobic conditions (**Figures 3, 6**). Because of low K_M_ of glycerol kinase toward glycerol, in the aerobic conditions, the major fraction of glycerol flows may through the respiratory route which GlpK is involved in (Kumar and Park, 2017). In addition, Voegele et al. (1993) reported that mutations in GlpK decreased glycerol transport in *E. coli* under aerobic conditions. We also observed the reduction of ethanol, one metabolite of oxidative pathway of glycerol metabolism, in *K. pneumoniae* overexpressing *KPN00353*, compared to vector control (data not shown). Therefore, we hypothesize that inhibition of GlpK by KPN00353 leads to decrease in glycerol uptake under bacterial (micro)aerobic growth by unknown feedback mechanism. The decreased intracellular glycerol results in low activities of both oxidative and reductive pathways of glycerol metabolism in *K. pneumoniae* (**Figure 10**). In addition, Ashok reported that *glpK* mutant and wild-type *K. pneumoniae* demonstrated similar glycerol consumption and 1,3-PD production profiles under anaerobic condition with addition of nitrate as electron acceptor. The authors suggested the involvement of other kinases in glycerol metabolism (Ashok et al., 2013; Kumar and Park, 2017). Therefore, we suggest that overexpression of KPN00353 inhibits the functions of GlpK and other kinases playing similar roles in glycerol metabolism. Because 1,3-PD production by *K. pneumoniae* under microaerobic conditions has several benefits (Chen X. et al., 2003), the effects of the EIIA homolog or its cognate sugar on GlpK and 1,3-PD production under microaerobic conditions should be evaluated.

The gene *crr* encodes glucose-specific EIIA in *E. coli* and *S. enterica* serovar Typhimurium. Oh et al. (2013) reported that the production of 1,3-PD by the *K. pneumoniae crr* mutant strain in the presence of both glucose and glycerol in the medium is enhanced compared with the production of 1,3-PD by the parent strain. However, the production of 1,3-PD by the *crr* mutant and the parent strain in the medium containing glycerol without glucose was similar to the 1,3-PD production by the *crr* mutant strain in the presence of both glucose and glycerol (Oh et al., 2013). Many studies have developed various biotechnological processes, including process and genetic engineering approaches, to improve the production of 1,3-PD by microorganisms. Genetic engineering approaches include the overexpression of homologous or heterologous genes involved in the 1,3-PD synthesis pathway (Ma et al., 2010) and the deletion of genes involved in byproduct formation (Horng et al., 2010). Sen et al. (2015) reported that the addition of glucose to the glycerol fermentation led to increased cell mass but no improvement in the 1,3-PD production of *K. pneumoniae*. There are several EIIA homologs belonging to different families in *K. pneumoniae*.^2^ Our findings and the report by Oh et al. show that the deletion of EIIA does not enhance the 1,3 PD production of *K. pneumoniae* in medium containing glycerol without the cognate sugar (**Figure 3**) (Oh et al., 2013). However, we surmise that the KPN00353 mutant would retain a high level of 1,3-PD production in medium containing glycerol with the cognate sugar, such as the *crr* mutant in the medium containing glycerol with glucose (Oh et al., 2013).

The typical energies of salt bridge, hydrogen bond, and hydrophobic interactions are approximately 2 kcal/mol, 1 kcal/mol, and 0.7 kcal/mol, respectively. The pKa values of aspartate (D), glutamate (E), histidine (H) and arginine (R) side chains in unfolded protein are 3.9, 4.1, 6.0 and 12.5, respectively. However, the pKa values of aspartate and glutamate decrease in a folded protein, whereas the pKa values of histidine and arginine increase. Therefore, glutamate and aspartate are negatively charged residues, whereas arginine is a positively charged residue and histidine is a partially positively charged residue under the conditions of a protein pull-down assay. In contrast, with glutamate, aspartate and arginine, histidine provides weak affinity via salt-bridge and hydrogen-bond formation. As mentioned above, H65D, H65E and H65R KPN00353 mutants have stronger binding affinities with *Kp*GlpK than wild-type KPN00353. On the other hand, glutamine (Q) has an uncharged side chain, and therefore, H65Q has a weaker binding affinity with *Kp*GlpK than does wild-type KPN00353. Moreover, the H110Q KPN00353 mutant provides similar binding affinity to that of wild-type KPN00353 because residues H110 and Q110 provide hydrophobic interactions only with *Kp*GlpK.

Apart from the glucose family, there are as yet no reports of other EIIA homologues binding to GlpK. In this study, KPN00353, a member of the fructose-mannitol EIIA (EII^Fru^ and EIIA^Mtl^) family, was observed to bind to GlpK (**Figure 5**). By structure modeling and the construction of KPN00353 variants, we identified the important residues of EIIA in the fructose-mannitol EIIA family for protein binding and, hence, for the GlpK activity and 1,3-PD production (**Figures 6, 7, 8**).

## Author Contributions

W-YJ: performed the experiments, analyzed the data, wrote the manuscript and revised the work critically. NP: performed the experiments and analyzed the data. Y-TH: wrote the manuscript and revised the work critically. W-TC: performed the experiments and analyzed the data. C-CC: provide opinion. P-CS: conceived, designed and performed the experiments, analyzed the data, revised the work critically and procured funding.

## Conflict of Interest Statement

The authors declare that the research was conducted in the absence of any commercial or financial relationships that could be construed as a potential conflict of interest.

## Footnotes

1 http://www.pymol.org

2 http://www.genome.jp/kegg/kegg2.html
