# Supplementary material for: The Negative Effects of KPN00353 on Glycerol Kinase and Microaerobic 1,3-Propanediol Production in Klebsiella pneumoniae
Source: Front Microbiol. 2017 Dec 7;8:2441. doi: 10.3389/fmicb.2017.02441 (PMC5770620; doi:10.3389/fmicb.2017.02441)
Supplement: Supplementary file 1 [file Table_1.DOCX]

Table S1. primers used in this study

| primer | Sequence (5’🡪3’) | Purpose |
| --- | --- | --- |
| Gap353-352F | GAATGATGGCGACATGACA | junction i (Fig.2) |
| Gap353-352R | ACTGGCGGGTTCATTACTTG | junction i (Fig.2) |
| Gap352-351F | AGGAATCCACCGATCAGGA | junction ii (Fig.2) |
| Gap352-351R | CTGTCATGTCGCCATCATTC | junction ii (Fig.2) |
| Gap351-350F | GTTAAGCGATTGACCAGCAC | junction iii (Fig.2) |
| Gap351-350R | CTCGGGTTTGAAAACAGCAC | junction iii (Fig.2) |
| Gap350-349F | GGCGGCGAGGCAATCCAC | junction vi (Fig.2) |
| Gap350-349R | ACTGGCGGCCTGGACTGAC | junction vi (Fig.2) |
| Gap349-348F | CGCTCTTTGGCTCAGAGCAGC | junction v (Fig.2) |
| Gap349-348R | GATGCTGGCGCTGGACGTGCC | junction v (Fig.2) |
| pGEX-glpK-FP | TTTAAATGACCGACAAAAAATATATCG | GST-GlpK recombinant protein |
| pGEX-glpK-RP | GTCGACTTACGCTTCGTCGTGCTCTT | GST-GlpK recombinant protein |
| MrkD-SalI-FP | GTCGACTTAATCGTACGTCAGGTT | His-MrkD recombinant protein |
| MrkD-EcoRI-RP | GAATTCAATGTCGCTGAGGAAATTACTA | His-MrkD recombinant protein |
| 353 F | CGCCGAATATGTCAACGAGA | mutant KO353 construction |
| 353 R | ATATGACTGTCAGCATCGGC | mutant KO353 construction |
| pET30b_353_FP | GTCGACCTACTCCATTTGTTTTTGAATG | His-KPN00353 recombinant protein |
| pET30b_353_RP | GTCGACATGGAAATTATTTTTGACCCGT | His-KPN00353 recombinant protein |
| GcoA H65^-^ FP | ATTGCCATGCCGCAAGCCCGGCCGGAA AAA | mutated His-KPN00353(H65Q) protein |
| GcoA H65^-^ RP | TTTTTCCGGCCGGGCTTGCGGCATGGCAAT | mutated His-KPN00353(H65Q) protein |
| KPN353-RP-H65E | GATGACGGG ATTGCCATGCCGGAAGCCCGG CCGGAA AAAGGGGCA | mutated His-KPN00353(H65E) protein |
| KPN353-FP-H65E | AGCCCCTTTTTCCGGCCGGGCTTCCGG CATGGCAATCCCGTCATC | mutated His-KPN00353(H65E) protein |
| KPN353-RP-H65D | GATGACGGGATTGCCATGCCGGATGCCCGG CCGGAA AAAGGGGCA | mutated His-KPN00353(H65D) protein |
| KPN353-FR-H65D | AGCCCCTTT TTCCGGCCGGGCATCCGGCAT GGCAATCCCGTCATC | mutated His-KPN00353(H65D) protein |
| KPN353-RP-H65R | GATGACGGGATTGCCATGCCGCTGGCCCGGCCGGAAAAAGGGGCA | mutated His-KPN00353(H65R) protein |
| KPN353-FR-H65R | TGCCCCTTTTTCCGGCCGGGCACGCGGCATGGCAATCCCGTCATC | mutated His-KPN00353(H65R) protein |
| GcoA H110^-^ FP | GATGCTGACAGTCAGATCAAA ATGATCCAA | mutated His-KPN00353(H110Q) protein |
| GcoA H110^-^ RP | TTGGATCATTTTGATCTGACTGTCAGCATC | mutated His-KPN00353(H110Q) protein |
|  |  |  |
